# *ZNF460* Promotes *GSDME*-Driven Pyroptosis via *PKM2* Transcriptional Activation in Aortic Dissection

**DOI:** 10.31083/RCM48463

**Published:** 2026-03-18

**Authors:** Quanlin Yang, Xin Shao, Zihe Zheng, Mingliang Li, Changbo Xiao, Bo Chen, Guowei Tu, Ben Huang, Xiaofu Dai

**Affiliations:** ^1^Department of Cardiovascular Surgery, Fujian Medical University Union Hospital, 350000 Fuzhou, Fujian, China; ^2^Key Laboratory of Cardio-Thoracic Surgery (Fujian Medical University), Fujian Province University, 350108 Fuzhou, Fujian, China; ^3^Department of Cardiac Surgery, Zhongshan Hospital, Fudan University, 200032 Shanghai, China; ^4^Department of Cardiovascular Surgery, The General Hospital of Ningxia Medical University, 750003 Yinchuan, Ningxia Hui Autonomous Region, China; ^5^Department of Cardiovascular Surgery, Chest Hospital of Zhengzhou University, 450008 Zhengzhou, Henan, China; ^6^Department of Cardiovascular Surgery, Gaozhou People’s Hospital, 525200 Gaozhou, Guangdong, China; ^7^Department of Cardiac Intensive Care Centre, Zhongshan Hospital, Fudan University, 200032 Shanghai, China

**Keywords:** *ZNF460*, *PKM2*, *GSDME*, pyroptosis, aortic dissection

## Abstract

**Background:**

Aortic dissection (AD) is a cardiovascular emergency with high mortality; however, the underlying molecular pathophysiology of AD remains incompletely understood. Pyroptosis, a proinflammatory form of programmed cell death, contributes to vascular injury; nonetheless, the upstream transcriptional regulation of pyroptosis in AD is similarly poorly defined.

**Methods:**

Differentially expressed genes were identified in aortic tissues from AD patients (Gene Expression Omnibus (GEO) datasets) using bioinformatics analyses, with a focus on cell death-related candidates. *In vivo* AD mouse models and *in vitro* vascular smooth muscle cell (VSMC) systems were employed to investigate the roles of these genes in AD. Potential transcription factors for *pyruvate kinase M2* (*PKM2*) were predicted using the Just Another Simple Array Retrieval/Simple API for Repository (JASPAR) and University of California, Santa Cruz (UCSC) databases, and validated by luciferase reporter and chromatin immunoprecipitation assays. Gain- and loss-of-function approaches were used to dissect the zinc finger protein 460 (ZNF460)–PKM2–gasdermin E (GSDME) axis and the associated impact on pyroptosis and AD progression.

**Results:**

*PKM2* expression was markedly elevated in AD tissues. *PKM2* silencing suppressed GSDME cleavage, attenuated VSMC pyroptosis, and mitigated experimental AD, whereas *PKM2* overexpression aggravated these outcomes. GSDME upregulation rescued pyroptosis in *PKM2*-depleted cells. Mechanistically, the transcription factor ZNF460 directly bound to the *PKM2* promoter, enhancing *PKM2* transcription and activating downstream GSDME-mediated pyroptosis. *ZNF460* knockdown reduced pyroptotic cell death and preserved aortic wall integrity* in vivo*.

**Conclusions:**

This study identifies ZNF460 as a novel upstream regulator of *PKM2* that drives GSDME-dependent pyroptosis, thereby exacerbating AD progression. Targeting the ZNF460–PKM2–GSDME axis may represent a promising therapeutic strategy for preventing pyroptosis-driven vascular damage in AD.

## 1. Introduction

Aortic dissection (AD) is a catastrophic vascular disorder characterized by intimal tearing of the aorta and formation of a false lumen, often leading to acute organ ischemia, cardiac tamponade, and sudden death [1, 2]. Despite advances in imaging, surgical techniques, and endovascular therapies, its incidence and mortality remain high [3]. Although factors such as hypertension, connective tissue disorders, and medial degeneration have been implicated [4, 5], the molecular mechanisms driving AD progression remain incompletely defined, limiting the development of effective targeted therapies.

Pyroptosis is an inflammatory form of programmed cell death marked by membrane pore formation, cytoplasmic swelling, and release of pro-inflammatory cytokines such as interleukin-1 beta (IL-1β) and IL-18 [6, 7]. This process is driven primarily by the gasdermin protein family, particularly Gasdermin E (*GSDME*), whose N-terminal fragment forms membrane pores following proteolytic cleavage. Pyroptosis of vascular smooth muscle cells (VSMCs) compromises the structural integrity of the aortic wall [8, 9], whereas pyroptosis of infiltrating immune cells increases local inflammation, both of which accelerate rupture of the aortic wall [10, 11]. Although pyroptosis has been linked to various cardiovascular pathologies, the upstream pathways involved in pyroptosis in AD remain poorly understood.

Pyruvate kinase M2 (*PKM2*), a key glycolytic enzyme, has emerged as a multifunctional protein involved in metabolic reprogramming, inflammation, and regulation of cell death [12, 13, 14]. *PKM2* has been shown to promote pyroptotic cell death in several diseases, yet its role in AD is unknown [15, 16, 17]. Of particular interest is whether *PKM2* is linked to changes associated with pyroptosis in VSMCs during the progression of AD.

In this study, using integrative bioinformatics analyses and experimental validation, we identified zinc finger protein 460 (*ZNF460*), a zinc finger transcription factor, as a novel upstream regulator of *PKM2*. We demonstrate that *ZNF460* directly binds the *PKM2* promoter, activating *PKM2* expression, which in turn promotes *GSDME* cleavage and pyroptosis. This *ZNF460*–*PKM2*–*GSDME* axis accelerates VSMC destruction and exacerbates AD progression* in vivo*. These findings uncover a previously unrecognized transcriptional mechanism driving pyroptosis in AD and suggest potential therapeutic targets for this lethal disease.

## 2. Methods

### 2.1 Bioinformatics Analysis

We analyzed gene expression data from the GSE52093 and GSE153434 public databases to identify differentially expressed genes (DEGs) between AD patients and healthy controls. We performed a Venn analysis to intersect DEGs with pyroptosis-related genes (from GeneCards).

### 2.2 Tissue Samples

The aortic wall specimens from AD patients were obtained. Control samples were collected from the macroscopically intact, non-dissected portion of the ascending aorta in the same patients. After resection, some specimens were rapidly frozen in liquid nitrogen and stored at –80 °C, while others were fixed in 4% paraformaldehyde (Sigma-Aldrich, St. Louis, MO, USA) and then preserved in paraffin. All surgeries were performed at Fujian Medical University Union Hospital, and all patients were informed of the purpose of the study and provided written informed consent. This study was approved by the Fujian Medical University Union Hospital Ethics Committee (2024KY180). The study adhered to the Declaration of Helsinki guidelines.

### 2.3 Animal Experiments

Male C57BL/6 mice, aged 3 weeks and weighing 10–12 g, were used in these experiments. They were housed in a specific pathogen-free environment with a temperature of 22–28 °C and relative humidity of 50–60%. The animal experiments were approved by the Ethics Committee of Fujian Medical University Union Hospital (Approval Number: IACUC FJMU 2024-0275). All procedures were performed in accordance with the animal welfare guidelines and regulations. Mice (male C57BL/6, 3 weeks old) were housed under specific pathogen-free conditions with controlled temperature and humidity, and all efforts were made to minimize suffering. Mice were randomly divided into six groups: control, model, model + si-NC, model + si-*PKM2*, model + vector, and model + *PKM2*, with 6 mice per group included in the final analysis. Adenovirus-associated virus (AAV) vectors were used to deliver siRNA or overexpression constructs targeting PKM2. Specifically, the AAV9 serotype was chosen due to its high tropism for VSMCs, ensuring efficient gene transfer to the target population. Mice were injected via the tail vein with a total of 1 × 10^11^ viral genomes (vg) per mouse. The AAV was administered 1 week prior to β-aminopropionitrile (BAPN) treatment to ensure sufficient gene expression and stable knockdown or overexpression of PKM2 throughout the modeling period. Following AAV administration, mice were treated with β-aminopropionitrile (BAPN; Sigma, St. Louis, MO, USA) via drinking water (1 g/kg/day) for 4 weeks. Afterward, the mice were anesthetized with intraperitoneal injections of 0.4% pentobarbital sodium (10 mg/kg) (Sigma-Aldrich, St. Louis, MO, USA), fixed on the operating table, and the hair between the two scapulas was removed, and the skin was disinfected with povidone-iodine. A small 0.5 cm incision was made to create a pouch, and a miniature osmotic pump containing Angiotensin II (Ang Ⅱ, 1 µg/kg per min) (Sigma-Aldrich, St. Louis, MO, USA) was implanted subcutaneously on the back of the mice (the control group received an equal amount of saline, while the other groups received Ang Ⅱ). After 48 h, the mice were euthanized with intraperitoneal injections of 5% pentobarbital sodium (0.05 mL/10 g of body weight). The aorta was dissected from the aortic root to the left and right iliac arteries, marked, and divided. One segment was stored at –80 °C for subsequent mRNA and protein detection, and the other segment was fixed in paraformaldehyde for histological experiments.

### 2.4 Immunohistochemistry (IHC)

After routine dehydration, fixation, paraffin embedding, and serial sectioning, the paraffin sections were placed in a 65 °C oven for 1 h. The sections were dewaxed with three changes of xylene, rehydrated through a graded alcohol series, and rinsed with distilled water. Antigen retrieval was performed in a citrate buffer in a microwave oven, and the sections were incubated with 3% hydrogen peroxide (MACKLIN, Shanghai, China) for 20 min. After three washes with PBS (MACKLIN, Shanghai, China), the sections were blocked with 8% goat serum (Solarbio Life Sciences, Beijing, China) at room temperature for 1 h. The primary antibody was incubated overnight at 4 °C. The next day, the sections were washed three times with PBS, incubated with the secondary antibody for 30 min, and washed three more times with PBS. DAB staining was then performed, and the sections were photographed and examined under an optical microscope.

### 2.5 RT-PCR

In AD and normal aortic wall tissue samples, we used TRIzol reagent (Invitrogen, Carlsbad, CA, USA) to extract RNA from the tissue through phenol-chloroform extraction. We transcribed the RNA into cDNA using oligo (dT) primers and the Transcriptor First Strand cDNA Synthesis Kit (4896866001, Roche, Basel, Switzerland). GAPDH was used as an internal reference, and RT-PCR was performed on a Bio-Rad RT-PCR instrument (CFX96, Hercules, CA, USA) to amplify the target gene and the reference gene. We calculated and compared the relative expression levels in each tissue based on the Ct values. The PCR reaction system was 20 µL, using a two-step method, 5 min at 95 °C followed by 40 cycles, each cycle consisting of 5 sec at 95 °C and 30 sec at 60 °C.

### 2.6 Western Blot

In 100 mg of frozen aortic wall tissue, and after cutting and grinding, we extracted total protein from the aortic wall tissue using RIPA lysis buffer (P0013B, Beyotime Biotechnology, Shanghai, China). We measured the protein concentration using a BCA protein quantification kit (23225, ThermoFisher Scientific, Rockford, IL, USA). We performed SDS-PAGE electrophoresis with 24 µg of protein and transferred it onto a PVDF membrane (IPVH00010, Millipore, Bedford, MA, USA). The membrane was blocked with 5% non-fat milk at room temperature for 1 h, then incubated the membrane with the appropriate primary antibody dilution solution at 4 °C overnight. The next day, the membrane was washed 3 times with TBST (TBS containing 0.1% Tween 20, P0231, Beyotime Biotechnology, Shanghai, China), 5 min each time. Then, we added the corresponding alkaline phosphatase-labeled secondary antibody and incubated the tissue at room temperature for 1 h. We used a ChemiDoc^TM^ XRS+ imaging system (Bio-Rad, Hercules, CA, USA) to detect the protein signal after adding the developing solution.

### 2.7 HE Staining

After sectioning the paraffin blocks, we baked the slices at 65 °C for 1 h, then sequentially hydrated them through xylene, 100%, 95%, and 70% ethanol, and stained them using hematoxylin (5 min), 1% ethanol hydrochloride (1 s), Scott’s solution (1 min), and eosin (1 min). Finally, the samples were dehydrated through graded ethanol solutions (70%, 95%, and 100%), cleared in xylene, and mounted. The basic pathological changes were compared between AD and normal aortic wall samples under a microscope (CKX41, Olympus, Tokyo, Japan).

### 2.8 ELISA

The levels of alanine aminotransferase (ALT), aspartate aminotransferase (AST), IL-1β, and C-reactive protein (CRP) in the serum of mice in each group were detected to evaluate the inflammatory and metabolic status of AD using commercial ELISA kits (Cat Nos. [ALT: AB282882], [AST: AB263882], [IL-1β: AB197742], [CRP: AB222511]; Abcam, Cambridge, MA, USA).

### 2.9 Immunofluorescence

We used immunofluorescence to detect the expression of phenotype transformation markers, Alpha-smooth muscle actin (α-SMA) and smooth muscle protein 22-alpha (SM22α) in VSMCs to assess the effect of *PKM2* on VSMCs. The slices were baked at 65 °C for 2 h, deparaffinized in xylene, and rehydrated through a graded ethanol series (100%, 95%, 85%, 75%, and 50%). We washed the specimens with phosphate-tween buffer solution, and perforated the membrane with 0.2% Triton solution for 15 min. We then restored the tissue in a citrate buffer solution. After naturally cooling for 2 h, we blocked the tissue with goat serum for 30 min, added the primary antibody, and incubated at 4 °C overnight. The next day, we thawed the tissue at room temperature for 1 h the next day, washed it 3 times with phosphate-tween buffer solution, then added and incubated the secondary antibody for 1 h. We then washed it again 3 times, stained the nuclei with DAPI for 5 min, added an anti-fluorescence quenching mounting medium, and observed it under an Olympus IX83 fluorescence microscope (Olympus, Japan).

### 2.10 Cell Culture and Treatment

Primary mouse aortic VSMCs were purchased from SUNNCELL (SNL-521, Wuhan, China). Mouse aortic VSMCs were cultured in DMEM medium (Gibco, Thermo Fisher Scientific, Waltham, MA, USA) containing 10% fetal bovine serum (Gibco, Thermo Fisher Scientific, Waltham, MA, USA) at 37 °C in a 5% CO_2_ incubator. Cells were cultured according to the manufacturer’s instructions and used at passages 3–5 for all experiments. When the cell density reached 70% under the microscope, we starved the cells by treating them with serum-free medium for 12 h, and then divided the cells into 8 groups for treatment: si-NC, si-*PKM2*, Ang II + si-NC, Ang II + si-*PKM2*, as well as vector, *PKM2*, Ang II + vector, Ang II + *PKM2*.

### 2.11 Luciferase Reporter Assay

The binding of *ZNF460* to the *PKM2* promoter was predicted using the Just Another Simple Array Retrieval/Simple API for Repository JASPAR and University of California, Santa Cruz UCSC Genome Browser Home databases. The promoter sequence of *PKM2* and the *ZNF460* binding site were obtained from the JASPAR website. This promoter sequence and the mutant promoter sequence were cloned into the pGL4-Luc-Report vector (Promega, Shanghai, China). The constructed plasmids were then transfected into VSMCs. After transfection, the cells were divided into two groups: one group was transfected with the vector alone; the other group was transfected with pcDNA-*ZNF460*. The cells were cultured for 48 h after treatment, and luciferase activity was determined using a dual-luciferase reporter assay kit (Cat. No. E1910, Promega, Madison, WI, USA).

### 2.12 Chromatin Immunoprecipitation (ChIP) Assay

Cells were seeded in 100 mm dishes and cultured until the cell density exceeded 90%. Based on the volume of the medium, 37.5% formaldehyde was added to each dish to achieve a final concentration of 0.75%. The mixture was then incubated on a rocker at room temperature for 10 minutes to allow cross-linking. Next, 1.5 mL of 2.5 M glycine was added to the mixture, which was further incubated on a rocker at room temperature for 5 minutes to stop the cross-linking reaction. The cells were collected and resuspended in 750 µL of FA lysis buffer. Chromatin was fragmented by sonication, generating fragments ranging from 100 to 1000 base pairs. 25 µg of the cell lysate was incubated with 4 µg of primary antibody and 50 µL of pre-treated Protein G agarose beads for immunoprecipitation (IP). The mixture was rotated and incubated overnight at 4 °C. The beads were washed three times with wash buffer and once with elution buffer. 200 µL of elution buffer was added to the IP samples and rotated at room temperature for 15 min. The IP samples were then treated with RNase A and proteinase K, and DNA was extracted using the phenol-chloroform method. PCR was used to confirm the binding of *ZNF460* to the DNA region of the *PKM2* promoter.

### 2.13 Statistical Analysis

All experimental data were statistically analyzed using SPSS 22.0 (IBM Corp., Armonk, NY, USA), and the results were expressed as mean ± standard deviation. ANOVA with Tukey’s honestly significant difference (HSD) post hoc test was used for multi-factor comparisons, and an independent sample *t*-test was used for direct group comparisons. *p* < 0.05 was considered statistically significant. We have adhered to ARRIVE guidelines.

## 3. Results

### 3.1 PKM2 is Markedly Upregulated in the Aortic Wall of AD Patients and Enriched in VSMCs

**Fig. 1.**
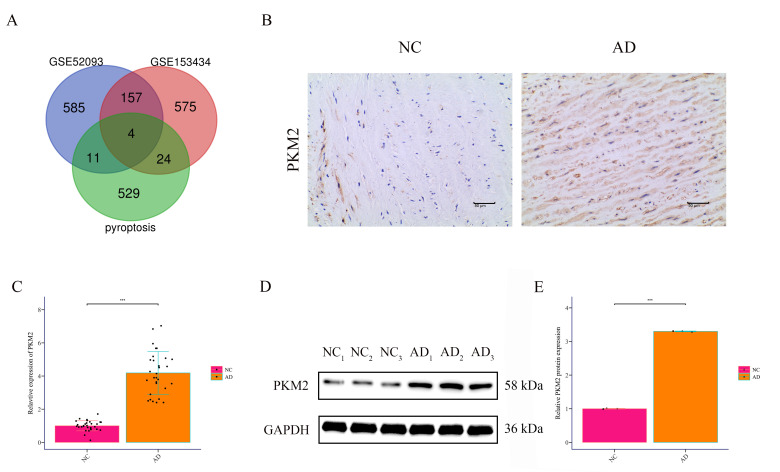
***PKM2* is highly expressed in the aortic wall tissues of patients with AD**. (A) Differential genes in AD patients and healthy controls from datasets GSE52093 and GSE153434 were analyzed and intersected with pyroptosis-related genes via Venn analysis. (B) Immunohistochemistry detected PKM2 expression in the aortic wall tissues of AD patients (AD) and non-dissected aortic tissues from the same patients (NC) (200×, scale bar = 50 μm). (C) RT-PCR measured *PKM2* expression in aortic wall tissues of AD patients (AD, n = 30) and non-dissected aortic tissues from the same patients (NC, n = 30). (D,E) Western Blot assessed PKM2 expression in aortic wall tissues of AD patients (n = 30) and non-dissected aortic tissues from the same patients (n = 30). ***p < 0.001. *PKM2*, pyruvate kinase M2; AD, aortic dissection; NC, Normal Control; RT-PCR, Reverse Transcription-Polymerase Chain Reaction.

Integrated analysis of two Gene Expression Omnibus (GEO) datasets (GSE52093, GSE153434) identified 161 differentially expressed genes in AD tissues compared with healthy controls. Intersection with pyroptosis-related genes yielded four candidates (*PLAUR*, *MLKL*, *PKM2*, *CXCL8*), among which *PKM2* displayed the most pronounced and specific upregulation (Fig. 1A). IHC confirmed elevated *PKM2* protein in AD aortic walls, particularly localized in vascular smooth muscle cells (VSMCs) (Fig. 1B). RT-qPCR further validated significantly higher *PKM2* mRNA expression in AD tissues (Fig. 1C), and Western Blotting confirmed increased PKM2 protein levels (Fig. 1D,E). These data established *PKM2* as a pyroptosis-associated molecule selectively upregulated in AD, especially within VSMCs.

### 3.2 PKM2 Promotes AD Progression and VSMC Pyroptosis In Vivo

**Fig. 2.**
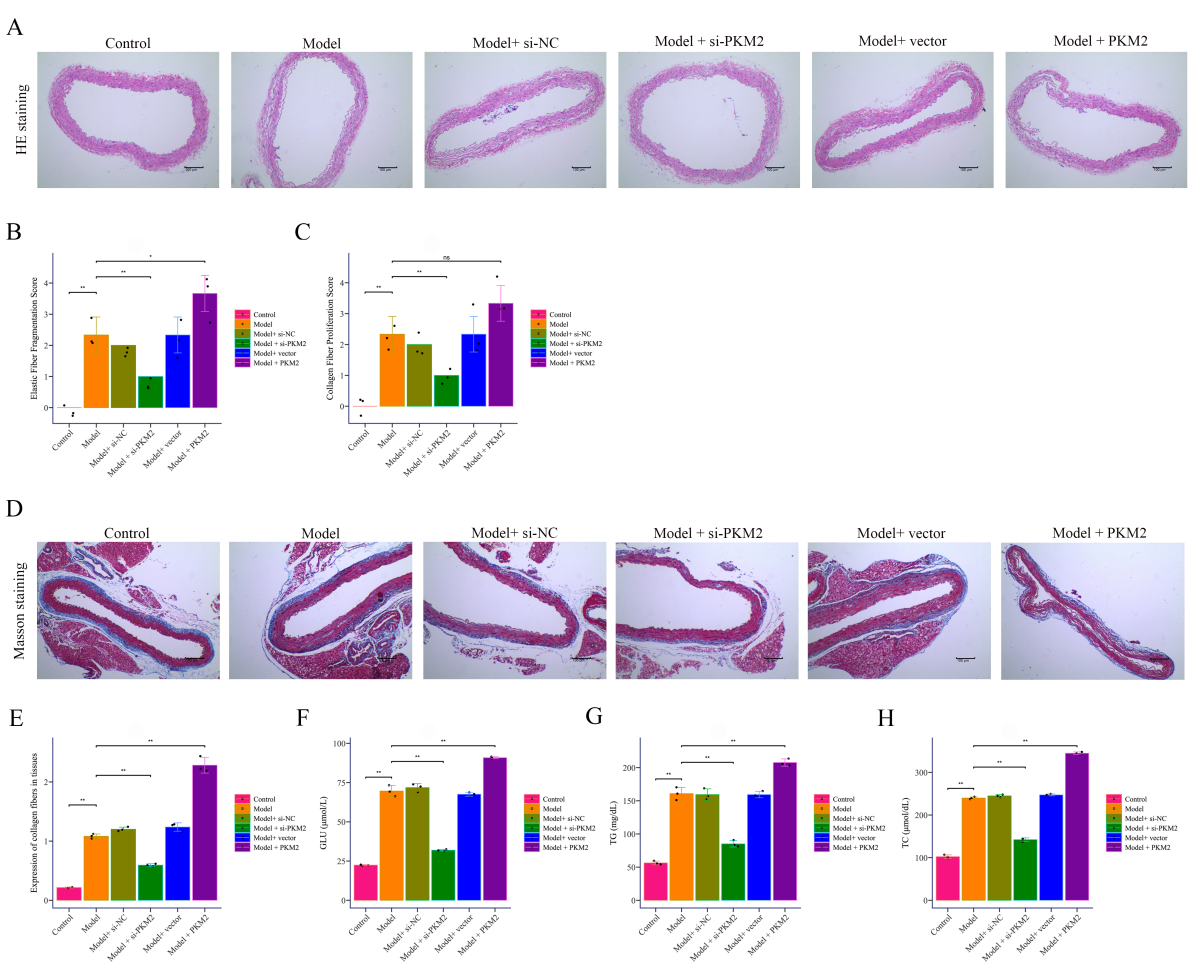
**The effect of *PKM2* on BAPN combined with Ang II-induced mouse AD**. (A–E) HE staining and Masson staining to detect pathological changes in AD (100×, scale bar = 100 μm). (F–H) The ELISA method to determine the effects of *PKM2* knockdown and overexpression on the levels of GLU, TC, and TG in the mouse AD model induced by BAPN combined with Ang II. *p < 0.05 *vs*. Model group, **p < 0.01 *vs*. Control group, *vs*. Model group, ns: no significant difference between groups. BAPN, β-aminopropionitrile; Ang II, Angiotensin II; GLU, Glucose; TC, Total Cholesterol; TG, Triglycerides.

**Fig. 3.**
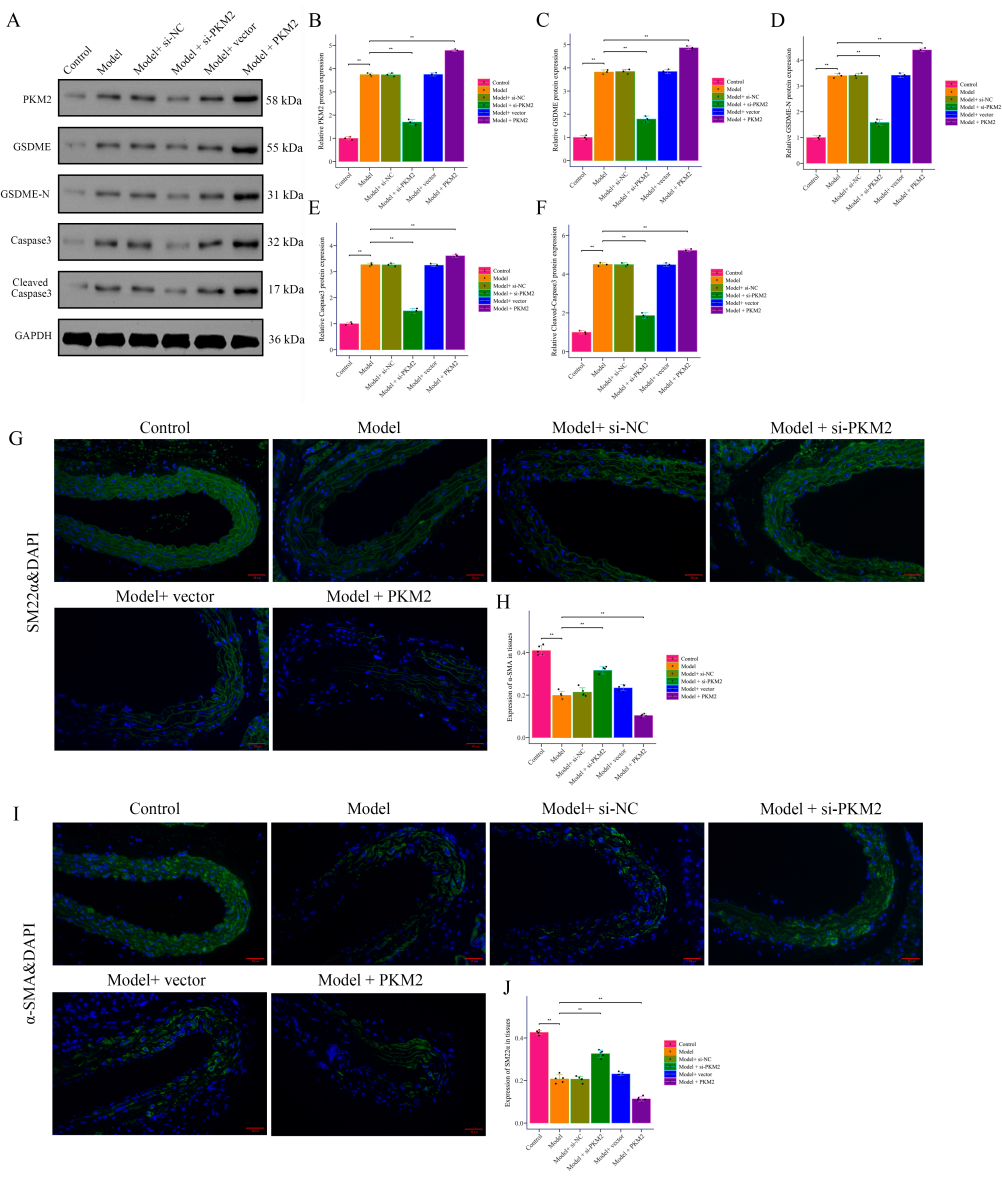
***PKM2* promotes pyroptosis in AD induced by BAPN combined with Ang II in mice**. (A–F) Western Blot was used to detect the expression of PKM2, GSDME, GSDME-N, caspase3, and cleaved caspase3 in the aortic wall tissue of mice with AD induced by BAPN combined with Ang II. (G–J) Immunofluorescence was used to detect the expression of α-SMA and SM22α (200×, scale bar = 20 μm). **p < 0.01. GSDME, Gasdermin E; α-SMA, Alpha-smooth muscle actin.

In the murine AD model, HE staining and Masson staining showed marked intimal tearing and intramural hematomas, which were mitigated by *Pkm2* knockdown (si-*Pkm2*) but exacerbated by *Pkm2* overexpression (Fig. 2A–E). Serum biochemical assays revealed increased glucose, total cholesterol, and triglycerides in AD mice, all attenuated by *Pkm2* silencing and aggravated by *Pkm2* overexpression (Fig. 2F–H). Western Blotting demonstrated elevated PKM2, GSDME, GSDME-N, and cleaved caspase3 in AD mice, reduced by *Pkm2* silencing and increased by overexpression (Fig. 3A–F). Loss of VSMC contractile markers α-SMA and SM22α in AD was reversed by *Pkm2* siRNA and decreased by *PKM2* overexpression (Fig. 3G–J). Thus, *PKM2* promotes AD pathology, enhances pyroptotic signaling, and drives VSMC phenotypic loss *in vivo*.

### 3.3 PKM2 Facilitates Ang II-Induced VSMC Phenotypic Transition and Pyroptosis

**Fig. 4.**
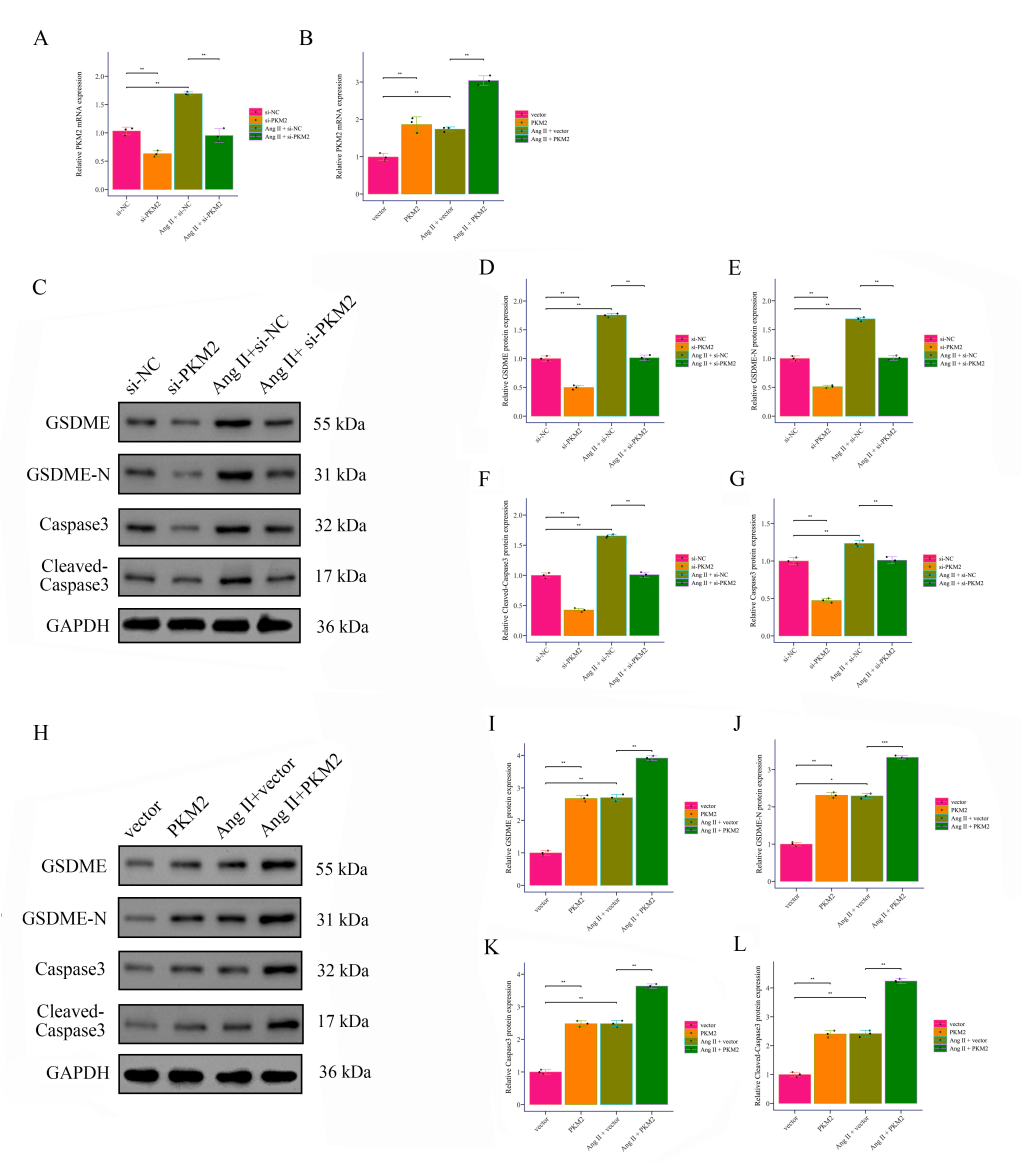
***PKM2* promotes Ang II-induced pyroptosis in VSMCs**. (A,B) The effects of *PKM2* downregulation and upregulation on *PKM2* expression in Ang II-induced and non-induced VSMCs were detected by RT-PCR. (C–L) The expression of pyroptosis markers GSDME, GSDME-N, caspase3, and cleaved caspase3 in Ang II-induced and non-induced VSMCs was detected by Western Blot. *p <0.05, **p < 0.01, ***p <0.001. VSMCs, vascular smooth muscle cells; si-NC, small interfering RNA negative control.

**Fig. 5.**
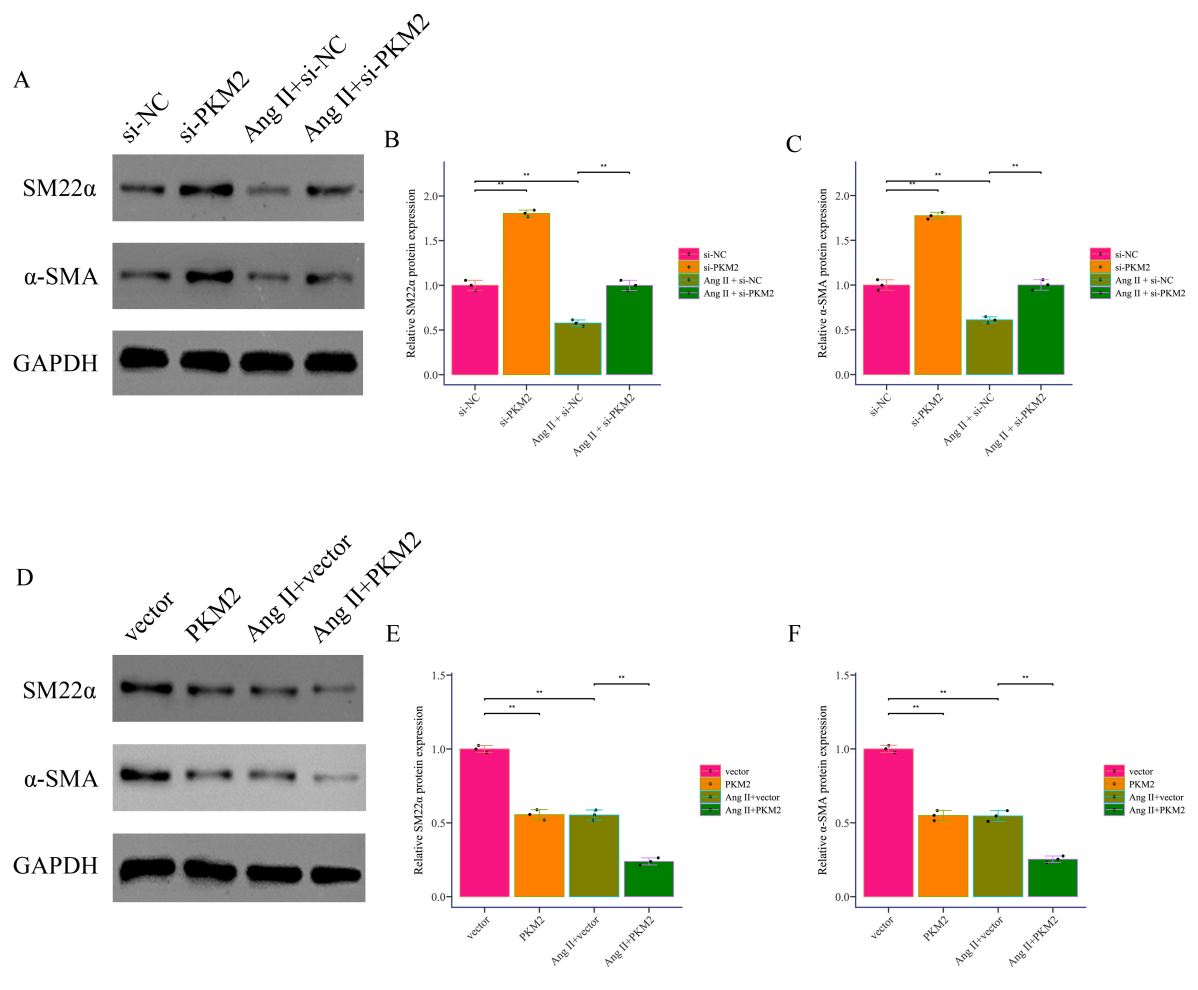
***PKM2* promotes Ang II-induced phenotypic transformation**. (A–C) The expression of SM22α and α-SMA, markers of VSMC phenotypic transformation, in Ang II-induced and non-induced VSMCs transfected with si-NC or si-*PKM2* was detected by by Western Blot. (D–F) The expression of SM22α and α-SMA, markers of VSMC phenotypic transformation, in Ang II-induced and non-induced VSMCs transfected with vector or PKM2 was detected by Western Blot. **p < 0.01.

The results showed that PKM2 expression was significantly increased in the Ang II treatment group, while si-PKM2 treatment significantly decreased PKM2 expression (Fig. 4A), and pcDNA-PKM2 treatment significantly increased PKM2 expression (Fig. 4B). Based on Western Blot results, the expression of GSDME, GSDME-N, and Cleaved-Caspase3 was significantly increased in the Ang II treatment group, while si-PKM2 treatment significantly reduced the expression of these proteins, and pcDNA-PKM2 treatment significantly increased the expression of these proteins (Fig. 4C–L). We measured lactate dehydrogenase (LDH) release in the supernatant of VSMCs under different treatment conditions. LDH release is a well-established indicator of membrane rupture and cell lysis, which are characteristic features of pyroptosis. Our results showed a significant increase in LDH release in the Ang II-treated group compared to the control group (**Supplementary Fig. 1A**). Notably, this increase was mitigated by PKM2 knockdown, suggesting that PKM2 plays a crucial role in Ang II-induced pyroptosis (**Supplementary Fig. 1A**). Using scanning electron microscopy (SEM) or high-magnification microscopy, we observed characteristic morphological changes in VSMCs treated with Ang II. Specifically, we detected membrane blebs and swelling, which are hallmark features of pyroptotic cell death (**Supplementary Fig. 1B**). These morphological changes were significantly reduced in cells where PKM2 was knocked down, further supporting the involvement of PKM2 in Ang II-induced pyroptosis (**Supplementary Fig. 1B**). The results of immunofluorescence staining indicated that SM22α and α-SMA expression were significantly decreased in the Ang II treatment group, while si-PKM2 treatment significantly enhanced α-SMA expression, and pcDNA-PKM2 treatment significantly reduced α-SMA expression (Fig. 5). These results suggest that PKM2 plays a crucial role in Ang II-induced cellular responses, and its inhibition can significantly alleviate Ang II-induced cellular responses, while its overexpression exacerbates this phenomenon.

### 3.4 PKM2 Promotes Pyroptotic Signaling Through GSDME

**Fig. 6.**
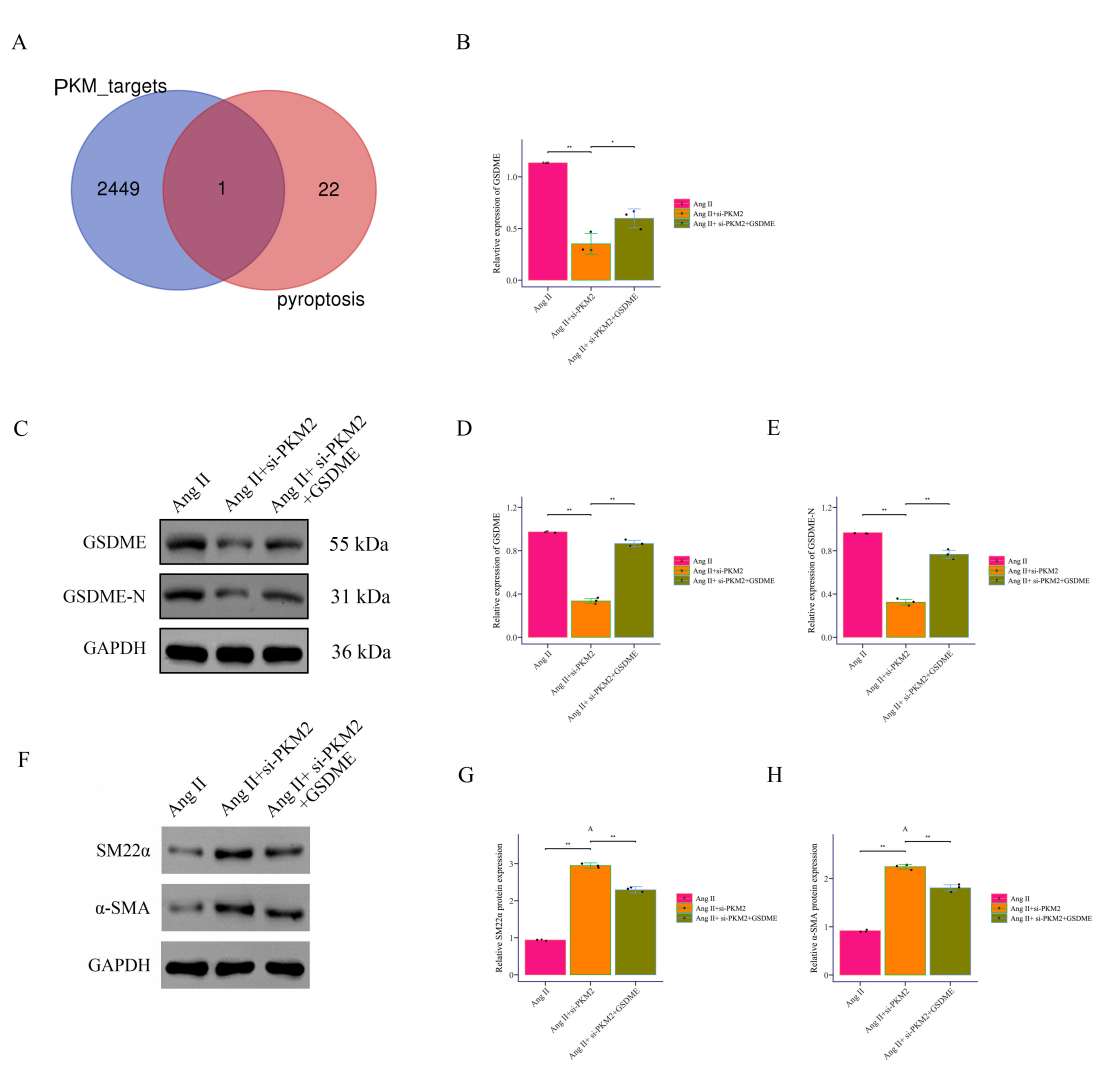
***PKM2* promotes phenotypic transformation and pyroptosis in Ang II-induced VSMCs by binding to *GSDME***. (A) Venn diagram analysis was used to identify the intersection of *PKM2* targets and pyroptosis targets. (B) GSDME expression was detected by RT-PCR. (C–E) GSDME and GSDME-N expression was detected by Western Blot. (F–H) The expression of SM22α and α-SMA was detected by Western Blot. *p < 0.05, **p < 0.01.

The intersection of *PKM2* targets and key pyroptosis proteins was analyzed by Venn analysis. The blue circle represents *PKM2* targets, and the red circle represents key pyroptosis genes. The overlapping part is the intersecting gene *GSDME* (Fig. 6A). *In vitro*, Ang II treatment significantly increased the expression of *GSDME*, while si-*PKM2* treatment significantly decreased the expression of *GSDME*, and overexpression of *GSDME* partially restored this effect (Fig. 6B). Western Blot analysis results showed that Ang II treatment significantly increased the expression of GSDME and GSDME-N, si-*PKM2* treatment significantly decreased the expression of these proteins, and overexpression of GSDME partially restored these changes (Fig. 6C–E). As Fig. 6F–H, the results demonstrated that compared to the Ang II group alone, si-*PKM2* treatment significantly increased SM22α and α-SMA expression, and overexpression of *GSDME* partially restored this phenomenon. These results indicate that *PKM2* participates in the regulation of Ang II-induced cellular responses through positively regulating the expression and activation of the GSDME.

### 3.5 ZNF460 Directly Activates PKM2 Transcription to Enhance GSDME-Mediated Pyroptosis

**Fig. 7.**
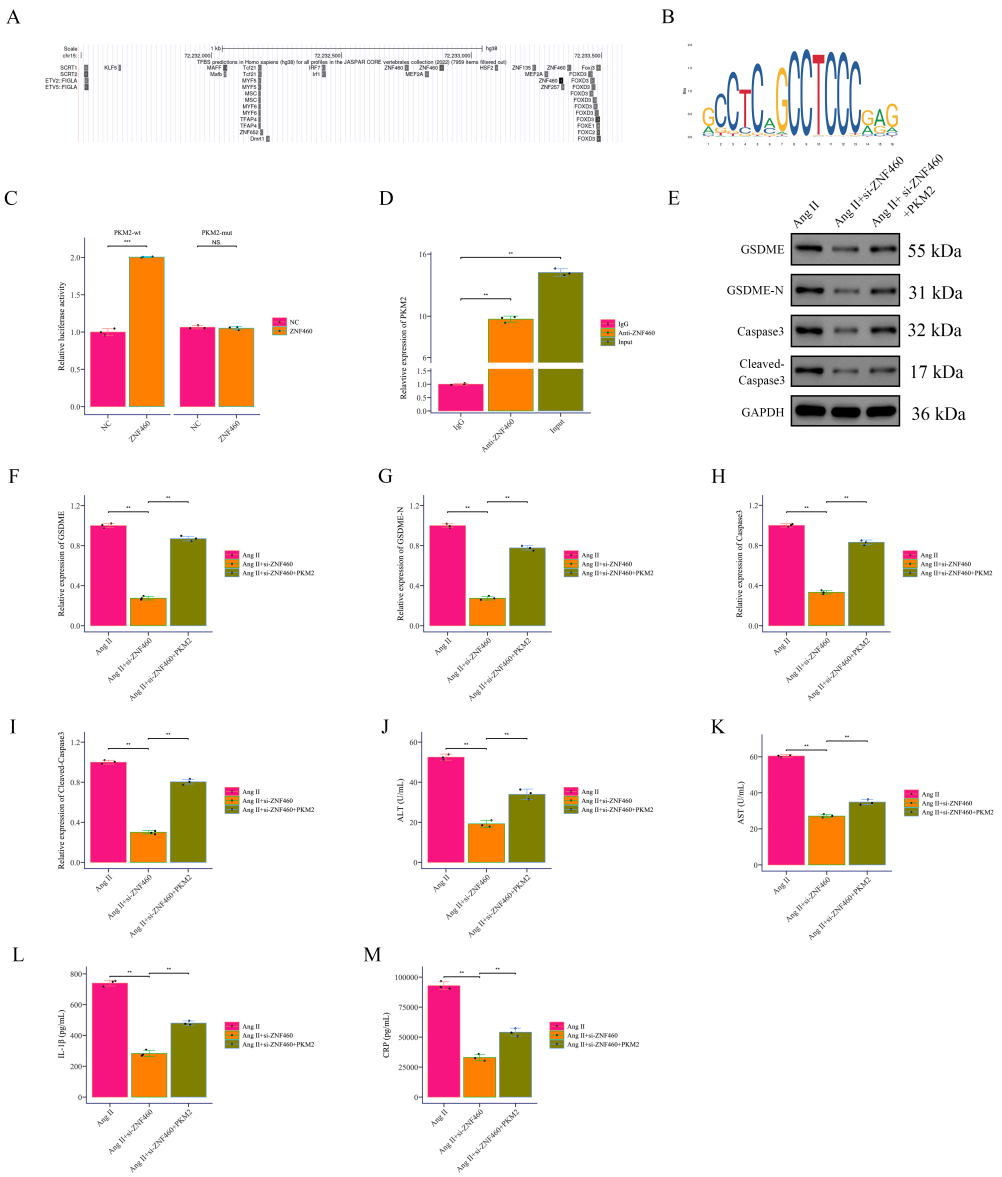
***ZNF460* promotes *PKM2* expression at the transcriptional level**. (A) Prediction of transcription factors near the *PKM2* promoter using the UCSC database. (B) Sequence logo of *ZNF460* DNA-binding motif obtained from the JASPAR database. The height of letters at each position indicates the nucleotide frequency in binding sites. (C) Luciferase reporter assay showing the effect of transcription factor *ZNF460* on *PKM2* expression. (D) ChIP-qPCR analysis of *ZNF460* binding to the *PKM2* promoter. (E–I) Western Blot detection of pyroptosis markers GSDME, GSDME-N, caspase3, and cleaved caspase3. (J–M) ELISA measurement of inflammatory factor markers ALT, AST, CRP, and IL-1β levels. **p < 0.01, ***p < 0.001. ns: no significant difference between groups. *ZNF460*, Zinc Finger Protein 460; UCSC, University of California, Santa Cruz; JASPAR, Just Another Simple Array Retrieval/Simple API for Repository; ALT, Alanine Aminottransferase; AST, Aspartate Aminottransferase; CRP, C-Reactive Protein.

Promoter analysis revealed putative *ZNF460* binding sites in the *PKM2* promoter (Fig. 7A,B). Luciferase assays demonstrated that *ZNF460* increased wild-type *PKM2* promoter activity but not mutant constructs (Fig. 7C). ChIP assays confirmed direct binding of *ZNF460* to the *PKM2* promoter in VSMCs (Fig. 7D). To validate the clinical relevance of *ZNF460* as a key upstream regulator in aortic dissection, we conducted additional experiments using human tissue samples. We performed quantitative real-time polymerase chain reaction (qRT-PCR) and IHC staining to assess the expression levels of ZNF460 in both AD tissues and non-dissected control tissues. The results of qRT-PCR demonstrated that *ZNF460* mRNA was significantly upregulated in AD tissues compared to non-dissected controls (**Supplementary Fig. 2A**). Consistent with the qRT-PCR data, IHC analysis revealed a marked increase in ZNF460 protein expression in AD tissues (**Supplementary Fig. 2B**). *ZNF460* silencing reduced GSDME, GSDME-N, caspase3, and cleaved caspase3 expression, whereas *PKM2* overexpression partially restored them (Fig. 7E–I). Correspondingly, Ang II–treated cells with *ZNF460* knockdown showed reduced serum ALT, AST, CRP, and IL-1β, which were reversed by *PKM2* overexpression (Fig. 7J–M). These results position *ZNF460* as a transcriptional activator of *PKM2*, amplifying *GSDME*-mediated pyroptosis in VSMCs and contributing to the pathophysiology of AD.

## 4. Discussion

In this study, we identified and characterized a previously unrecognized transcriptional axis, *ZNF460*–*PKM2*–*GSDME*, as a driver of VSMC pyroptosis and the progression of AD. Using integrated bioinformatics, *in vitro* experiments, and *in vivo* murine models, we demonstrated that *ZNF460* directly binds the *PKM2* promoter, enhancing its transcriptional activity. Elevated *PKM2* expression promoted caspase3 activation, *GSDME* cleavage, and pyroptotic cell death, resulting in the release of IL-1β and IL-18, medial degeneration, and exacerbated AD pathology. This work represents the first demonstration of a zinc finger transcription factor linking metabolic reprogramming to pyroptosis in VSMCs in AD, a mechanism not addressed in previous studies. Silencing either *ZNF460* or *PKM2* mitigated pyroptotic signaling, preserved VSMC phenotype, and attenuated aortic wall destruction. These findings provide novel mechanistic insight into the inflammatory tissue injury that drives AD and suggest a potentially treatable pathway for intervention.

Pyroptosis has emerged as a crucial link between cellular stress responses and inflammatory vascular injury. Pyroptosis, classically mediated by the gasdermin family proteins, is characterized by pore formation in the plasma membrane and efflux of pro-inflammatory cytokines, which amplify local immune activation [18]. In cardiovascular pathology, pyroptosis contributes to atherosclerotic plaque instability [19], myocardial ischemia-reperfusion injury [20], and abdominal aortic aneurysm (AAA) [21]. Our findings extend this paradigm to AD, revealing that *GSDME* is the principal driver of pyroptosis in VSMCs, consistent with evidence that *GSDME* is preferentially cleaved by caspase3 and bridges apoptotic and pyroptotic signaling [22]. The dominance of caspase3–dependent pyroptosis in our model suggests that classical apoptotic stimuli in AD, such as oxidative stress and biomechanical injury, may be rerouted towards inflammatory cell death, thereby increasing medial damage. Compared with macrophage or neutrophil-driven pyroptosis, VSMC pyroptosis directly undermines the structural integrity of the aortic wall, making its pathological consequences potentially more irreversible.

*PKM2* is increasingly recognized as a multifunctional metabolic enzyme that integrates glycolytic flux with transcriptional regulation of inflammatory mediators [23]. In immune cells, *PKM2* can dimerize and translocate to the nucleus, acting as a co-activator of hypoxia-inducible factor-1 alpha (*HIF-1α*) or signal transducer and activator of transcription 3 (*STAT3*) and promoting expression of pro-inflammatory genes, including *IL‑1β* and NOD-like receptor family pyrin domain containing 3 (*NLRP3*) [23]. In our study, *PKM2* amplified caspase‑3–dependent cleavage of *GSDME*, suggesting a potential mechanism whereby metabolic reprogramming primes cells for pyroptotic responses. This may involve enhanced glycolysis and lactate accumulation, accompanied by reactive oxygen species (ROS) production, which can further augment caspase3 activation and membrane pore formation. Given that VSMCs under hemodynamic overload often exhibit a metabolic shift toward glycolysis, *PKM2* upregulation may serve as a critical switch linking metabolic stress to inflammatory cell death. This is in line with reports where *PKM2* modulates both metabolic remodeling and cell death thresholds in cancer cells, implying a conserved regulatory role across different diseases [24].

While pyroptosis in macrophages has been extensively studied in vascular pathology [25], our results underscore that VSMCs themselves are highly susceptible to pyroptosis via the *ZNF460*–*PKM2*–*GSDME* axis. The destruction of contractile VSMCs not only weakens the aortic wall mechanically but also alters the extracellular matrix environment, potentially facilitating infiltration of inflammatory cells that undergo their own pyroptotic death. This creates a feed-forward loop of inflammation and tissue damage. Therapeutically, selectively modulating pyroptosis in VSMCs while preserving immune surveillance could be a sophisticated strategy for AD management, especially during the acute phase when rapid wall destabilization is imminent.

Beyond vascular disorders, *GSDME*-mediated pyroptosis has been implicated in chemotherapy-induced tissue injury, neurodegeneration, and septic organ damage in sepsis [26, 27, 28]. The recurrent theme across these contexts is that *GSDME* links apoptotic executioner caspase3 to the inflammatory outcomes of pyroptosis, particularly in non-immune parenchymal cells. Our findings suggest that the same coupling exists in VSMCs during AD, highlighting *GSDME* as a pivotal molecular switch that converts apoptotic cues into inflammatory cell death. Given ongoing efforts to develop inhibitors of *GSDME* cleavage or pore formation in oncology, there is an opportunity to accelerate their use for cardiovascular indications.

*ZNF460* is a zinc finger transcription factor with limited characterization in cardiovascular biology. Previous studies have implicated ZNFs in regulating inflammatory and apoptotic signaling [29], but a direct role in pyroptosis has not been reported. We demonstrate that *ZNF460* binds directly to the *PKM2* promoter and enhances its transcriptional activity. This positions *ZNF460* as a novel upstream driver of pyroptosis in VSMCs, expanding the functional repertoire of zinc finger proteins in vascular pathology. Given that transcription factors are generally considered challenging drug targets, future studies should explore indirect inhibition strategies, such as interfering peptides or small molecules disrupting *ZNF460*–DNA binding.

It is plausible that the *ZNF460*–*PKM2*–*GSDME* axis interacts with known inflammatory signaling hubs such as NLRP3 inflammasomes, MAPK cascades, or nuclear factor kappa B (NF‑κB) activation. *PKM2*-mediated *STAT3* activation, for example, can enhance NF‑κB–dependent transcription and augment IL‑1β production [6]. Similarly, oxidative stress in AD can activate MAPK signaling and mitochondrial ROS generation, upstream activators of caspase‑3 [30]. Mapping these interactions could uncover additional interventions and clarify whether pyroptosis acts synergistically or competitively with ferroptosis and necroptosis in VSMCs.

The dual function of *PKM2*, as both a metabolic and a signaling regulator, underscores its therapeutic potential. Small‑molecule *PKM2* inhibitors (e.g., shikonin, compound 3K) have demonstrated anti‑inflammatory and anti‑tumor activities in preclinical settings [31, 32]. Disrupting *GSDME* cleavage or modulating caspase‑3 activation could mitigate pyroptotic damage without abolishing vital cell death pathways. Although transcription factors like *ZNF460* have traditionally been considered “undruggable”, advances in targeting protein-DNA interactions and RNA therapeutics make indirect modulation increasingly feasible. Moreover, *PKM2* expression in aortic tissue or potentially in circulating vesicles could serve as a biomarker for disease activity and response to therapy.

### Limitations

There are several limitations to this study. While our model recapitulates acute AD-like medial rupture, the chronic degenerative aspects seen in human AD may not be fully represented. It is important to acknowledge a limitation regarding the interpretation of PKM2 upregulation in human AD tissues. Since samples were obtained post-operatively, it is difficult to distinguish whether the increase in PKM2 was a primary driver of the dissection or a secondary response to the acute inflammatory storm following rupture. However, our *in vitro* data demonstrating that PKM2 knockdown mitigates Ang II-induced injury suggest that PKM2 plays a functional role in the pathogenesis, rather than being a mere consequence. We propose that PKM2 may be involved in a positive feedback loop where the initial vascular injury upregulates PKM2, which subsequently amplifies inflammatory responses and vascular smooth muscle cell death, further aggravating the disease. The transcriptional network of *ZNF460* is incompletely mapped; other pyroptosis- or metabolism-related target genes may exist. Co‑culture systems, including VSMCs and immune cells, or vessel‑on‑a‑chip models, could further clarify intercellular pyroptosis crosstalk. Future studies will also need to validate these findings in larger patient cohorts and across multiple centers to strengthen their translational potential. Lastly, the safety and efficacy of targeting the *ZNF460*–*PKM2*–*GSDME* axis should be established in large‑animal studies before clinical translation. Another limitation of this study is that we did not distinguish between VSMC dedifferentiation and cell death in the analysis of marker expression. Future studies using co-staining of contractile markers with pyroptosis markers are needed to clarify the temporal relationship between these events. These adjacent tissues may still exhibit subtle pathological changes compared to healthy aortas, and thus our comparisons reflect differences between diseased and less-affected tissues rather than healthy versus diseased states.

## 5. Conclusions

In summary, this study identifies a critical regulatory axis involving *ZNF460*–*PKM2*–*GSDME* in the pathogenesis of AD. *PKM2* promotes VSMC phenotypic switching and pyroptosis, accelerating disease progression, while *GSDME* exacerbates the inflammatory response. The modulation of *PKM2* or *GSDME*, or the inhibition of *ZNF460*, could offer promising therapeutic avenues for the treatment of AD and other vascular diseases driven by pyroptosis. Future studies are required to explore the translational potential of targeting this axis in preclinical and clinical settings.

## Data Availability

The data supporting the findings of this study are available from the corresponding author upon reasonable request.

## Abbreviations

AD, aortic dissection; VSMCs, vascular smooth muscle cells; *PKM2*, pyruvate kinase M2; *ZNF460*, zinc finger protein 460; *GSDME*, Gasdermin E; AAA, abdominal aortic aneurysm; DEGs, differentially expressed genes.

## Supplementary Material

Supplementary material associated with this article can be found, in the online version, at https://doi.org/10.31083/RCM48463.
