# Supplementary material for: ZNF460 Promotes GSDME-Driven Pyroptosis via PKM2 Transcriptional Activation in Aortic Dissection
Source: Rev Cardiovasc Med. 2026 Mar 18;27(3):48463. doi: 10.31083/RCM48463 (PMC13036547; doi:10.31083/RCM48463)
Supplement: Supplementary file 1 [file 2153-8174-27-3-48463-s1.zip › Supplementary Fig.1.docx]

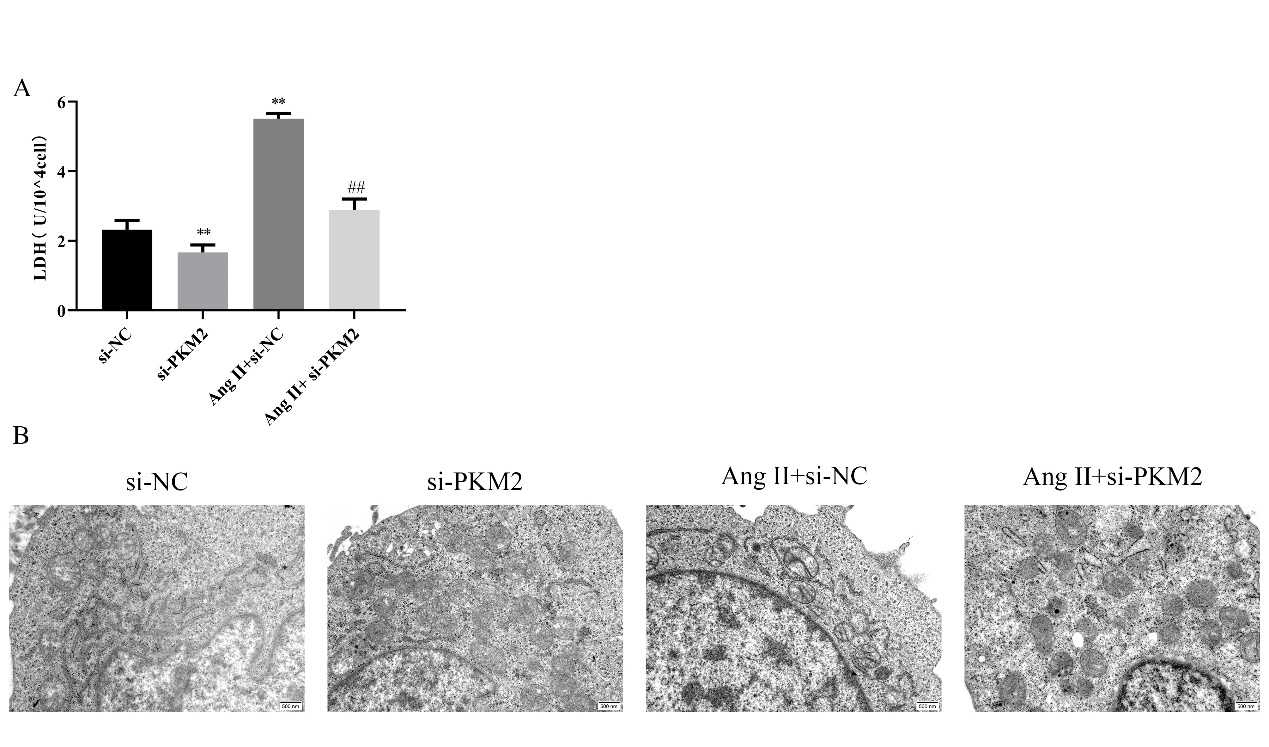


**Supplementary Fig. 1. The mitigation of LDH release and morphological changes by PKM2 knockdown.** (A) LDH release levels were detected by ELISA in VSMCs under different treatment conditions. (B) SEM images of VSMCs under different treatment conditions. ***P* < 0.01 *vs*. si-NC group, ^##^*P* < 0.01 *vs*. Ang II + si-NC.
