# Supplementary material for: ZNF460 Promotes GSDME-Driven Pyroptosis via PKM2 Transcriptional Activation in Aortic Dissection
Source: Rev Cardiovasc Med. 2026 Mar 18;27(3):48463. doi: 10.31083/RCM48463 (PMC13036547; doi:10.31083/RCM48463)
Supplement: Supplementary file 1 [file 2153-8174-27-3-48463-s1.zip › Supplementary Fig.2.docx]

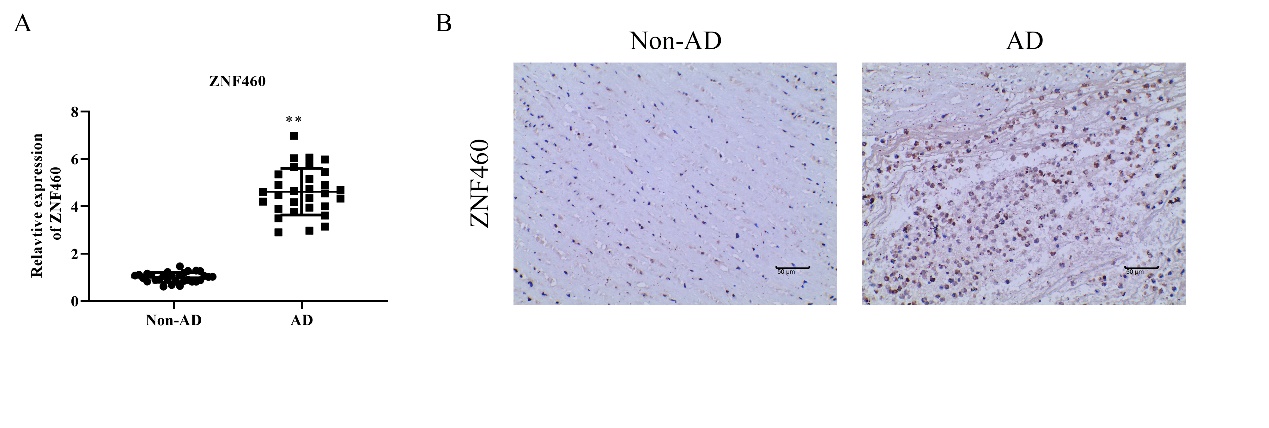


**Supplementary Fig. 2. ZNF460 expression levels in AD tissues.** (A) qRT-PCR analysis showing significantly higher ZNF460 mRNA expression in AD tissues compared to non-dissected controls. (B) IHC staining demonstrating increased ZNF460 protein expression in AD tissues. ***P* < 0.01 *vs*. Non-AD group.
