# Supplementary figures and images for: ZNF460 Promotes GSDME-Driven Pyroptosis via PKM2 Transcriptional Activation in Aortic Dissection
Source: Rev Cardiovasc Med. 2026 Mar 18;27(3):48463. doi: 10.31083/RCM48463 (PMC13036547; doi:10.31083/RCM48463)

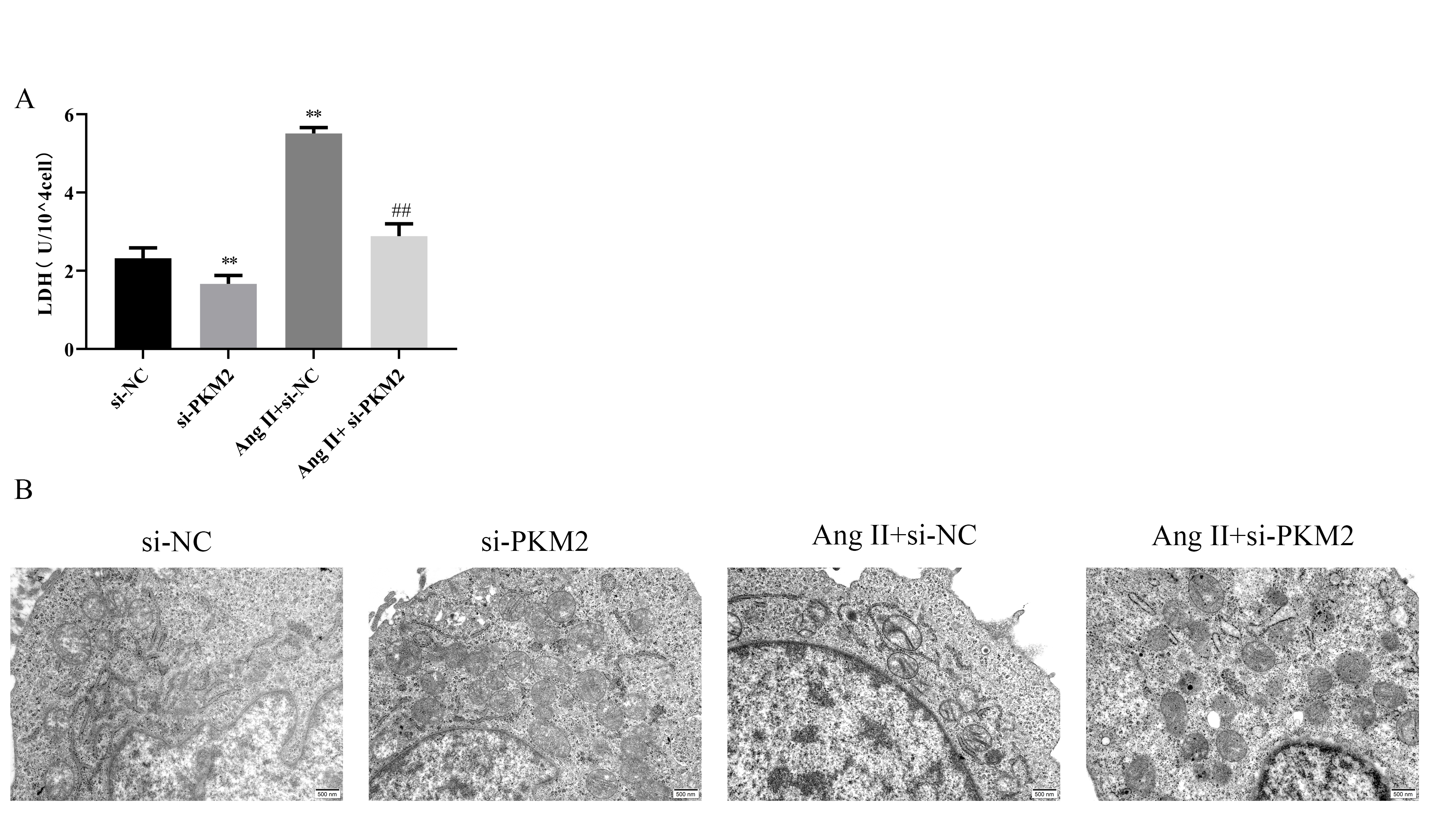

Supplement: Supplementary file 1 [file 2153-8174-27-3-48463-s1.zip › Supplementary Fig.1.jpg]

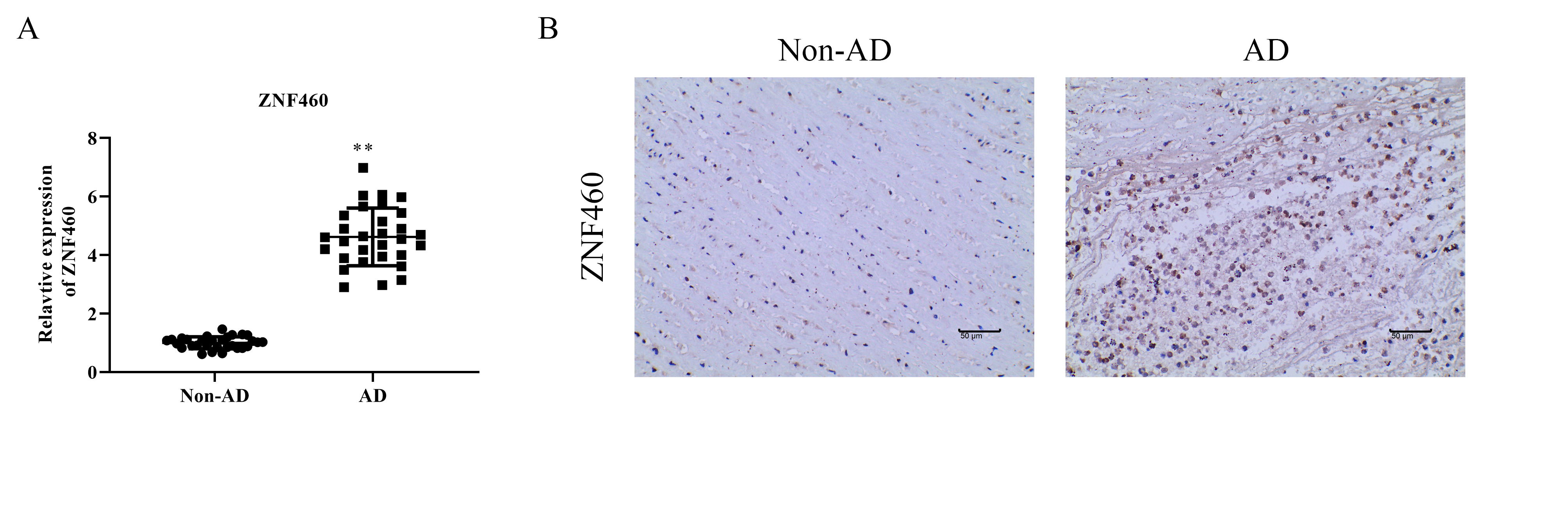

Supplement: Supplementary file 1 [file 2153-8174-27-3-48463-s1.zip › Supplementary Fig.2.tif]
